# Unveiling the Effects of Copper Ions in the Aggregation of Amyloidogenic Proteins

**DOI:** 10.3390/molecules28186446

**Published:** 2023-09-05

**Authors:** Valentina Oliveri

**Affiliations:** Dipartimento di Scienze Chimiche, Università degli Studi di Catania, Viale A Doria 6, 95125 Catania, Italy; valentina.oliveri@unict.it; Tel.: +39-095-738-5094

**Keywords:** copper, amyloid, misfolding, Alzheimer, Parkinson, prion, diabetes, synuclein, tau, Neurokinin B

## Abstract

Amyloid diseases have become a global concern due to their increasing prevalence. Transition metals, including copper, can affect the aggregation of the pathological proteins involved in these diseases. Copper ions play vital roles in organisms, but the disruption of their homeostasis can negatively impact neuronal function and contribute to amyloid diseases with toxic protein aggregates, oxidative stress, mitochondrial dysfunction, impaired cellular signaling, inflammation, and cell death. Gaining insight into the imbalance of copper ions and its impact on protein folding and aggregation is crucial for developing focused therapies. This review examines the influence of copper ions on significant amyloid proteins/peptides, offering a comprehensive overview of the current understanding in this field.

## 1. Introduction

In recent times, the increasing prevalence of various amyloid diseases has emerged as a global cause for concern [1,2]. Amyloidosis can develop either from a normal native protein that assumes an altered arrangement, known as sporadic amyloidosis, or from a protein with mutations, which is referred to as familial or hereditary amyloidosis [2].

Misfolding processes in the human body can lead to the formation of stable protein aggregates called amyloid fibrils, which are associated with several disorders (there are more than 30 known amyloid-related diseases) including Alzheimer’s disease (AD), Parkinson’s disease (PD), Type II diabetes mellitus (T2DM), Huntington’s disease (HD), prion disease, etc. [2]. Each of these disorders is linked to a particular peptide or protein. However, the amyloid fibrils exhibit common structural characteristics (high β-sheet content) across different pathologies. The current understanding suggests that the misfolding of polypeptide chains is a shared characteristic in these diseases, competing with the normal folding pathway. Recent studies on amyloidogenic proteins indicate that the most harmful species for living systems are prefibrillar aggregates that possess some levels of order, rather than fully formed and structured amyloid fibrils. These findings emphasize the significance of investigating the early stages of protein aggregation to comprehend the mechanisms underlying amyloid diseases.

A growing body of evidence suggests that transition metals, specifically in their divalent and trivalent ionic forms, can expedite the aggregation process of different pathological proteins [3,4]. This aspect is of particular relevance considering the evident alterations of metal ion concentrations in these pathologies. Metal ions, including copper, play prominent roles in various physiological processes within the brain and in other tissues, such as neurotransmission, enzyme activity, and antioxidant defense [5]. However, when their homeostasis is disrupted, it can lead to detrimental effects on cellular function and contribute to the progression of amyloidosis [6].

Imbalances in metal ion homeostasis can result in the formation of toxic protein aggregates, mitochondrial dysfunction, oxidative stress, impaired cellular signaling, and inflammation. These processes can ultimately lead to cell death and the characteristic symptoms of amyloid diseases. Moreover, emerging evidence suggests that restoring metal ion homeostasis through metal-binding therapy or the modulation of metal-related proteins could be a potential therapeutic approach for amyloidosis [7]. However, further research is needed to fully elucidate the complex interactions between metal ions and amyloidosis and to develop effective interventions.

Understanding the mechanisms underlying the dyshomeostasis of copper ions in amyloid diseases and its effects on protein folding/misfolding and aggregation is essential for developing targeted therapeutic strategies.

In this review, the effects of copper ions on the major amyloid proteins will be analyzed to provide an overview to the reader of the current knowledge on this topic.

## 2. Factors Influencing Protein Aggregation

Protein aggregation more often results from incorrect interactions with metal ions, local alterations in environmental conditions (such as pH, ionic strength and temperature, etc.), or chemical modifications (such as oxidative or proteolytic processes). Several environmental factors can affect the aggregation process. Experimental studies have demonstrated that even slight variations in these environmental factors can have a significant impact on the outcomes. The pH of the environment plays a crucial role in determining the type and density of the surface charge on proteins as well as the extent of structural disruption. Additionally, pH has an impact on the intramolecular folding of proteins and influences protein–protein interactions. 

It is important to note that the primary structure of the polypeptide influences its ability to convert into amyloid structures under specific conditions. Certain amino acid sequences, particularly those containing regions with a high propensity for β-sheet formation, are more prone to undergo conformational changes and aggregate into amyloid structures. For example, aggregation can occur through partially unfolded intermediates and unfolded states, such as protein translocation across membranes, or protein self-association. Polypeptides that are partially unfolded contain hydrophobic segments and demonstrate higher flexibility than when they are folded, which renders them more susceptible to the process of aggregation. 

The binding of copper, for example, to amyloidogenic proteins significantly affects protein toxicity by affecting either the aggregation process or the generation of radicals [8]. As for Cu, it is crucial to understand that its primary redox state differs between the intracellular and the extracellular environment. Cu is predominantly found in its reduced form, Cu^+^, inside the cell because of the reducing conditions. Conversely, in extracellular spaces, Cu^2+^ is more prevalent. Therefore, the significance of one redox state over the other depends on the location of the amyloidogenic peptide. Unlike the coordination of Cu^+^ ions, which typically involves atom donors found in side chains of amino acids such as methionine (M) or histidine (H), Cu^2+^ ions attach to the N-terminal amine or the imidazole group of H, followed by coordination with backbone amide nitrogen atoms. However, this coordination interaction is hindered when the protein assumes an α-helical conformation, as observed in the case of PrP, Aβ, and αSyn when interacting with membranes [9]. The interaction with the membrane, as in the case of αSyn, could modulate the conformational and aggregation properties of the proteins and also silence the metal-mediated redox reactivity of the metal–protein complexes [10]. 

To gain a comprehensive understanding of the intricate molecular processes and structural changes induced by copper ions in protein aggregation, researchers employ a variety of chemical and physical methods. The kinetics of amyloid aggregation is usually studied through ThT-based fluorescence experiments, while circular dichroism (CD), Nuclear Magnetic Resonance (NMR), and Molecular Dynamics (MD) simulations offer insights into the structural modifications occurring during the process, providing atomic-resolution structures of the species. To examine the size and morphologies of metal–protein aggregates, Dynamic Light Scattering (DLS) and Transmission Electron Microscopy (TEM)/Atomic Force Microscopy (AFM) images are utilized, respectively. Additionally, other methods such as Isothermal Titration Calorimetry (ITC), Mass spectrometry (MS), Electron Paramagnetic Resonance (EPR), and Fourier Transform Infrared Spectroscopy (FTIR) are used to investigate the interactions between metal ions and amyloidogenic peptides/proteins to obtain information on the metal-binding affinity and amino acid residues involved in metal complexation. Moreover, chemical tools play a crucial role in controlling the impact of metal–protein interactions on amyloid aggregation and toxicity, as well as in probing the aggregation pathways.

Various amyloidogenic peptides and their variants have been intensely investigated for their coordination sites with Cu^+^ or Cu^2+^, but not to an equal extent. Among these peptides, Aβ has been the most extensively examined, followed by αSyn and IAPP [11,12,13,14,15]. On the other hand, tau and other amyloid proteins have a smaller amount of information available regarding their interactions with metal ions [16,17,18]. The investigation of the metal complexes of these proteins has frequently been achieved using specific fragment peptides of the corresponding amyloid protein containing the metal-binding site, for example, Aβ_1–16_. Nevertheless, it is essential to acknowledge that in the case of short peptides like Aβ and IAPP, numerous studies have been conducted not only on the metal-binding domain but also on the full-length peptides. Similarly, for the longer αSyn, several studies have also focused on the full-length form. However, this approach does not apply to tau or other proteins that are significantly longer, and, for example in the case of tau, exist in various phosphorylate d states that could influence the metal coordination. If possible, this article will focus on presenting data acquired from the entire peptide or protein, as it is believed to better mimic real conditions.

### 2.1. α-Synuclein

α-Synuclein (αSyn) is a protein of 140 amino acids that is primarily found in the brain, particularly in regions involved in regulating movement, such as the substantia nigra. It is a key player in neurodegenerative diseases, particularly PD and dementia with Lewy bodies (DLB). Abnormal accumulation of αSyn in the form of insoluble aggregates is a hallmark pathology of these disorders [19]. In PD, the Lewy bodies are involved in the disruption of normal cellular processes and lead to the degeneration of dopamine-producing cells in the brain, causing the characteristic motor symptoms of PD [20].

**Figure 1:**
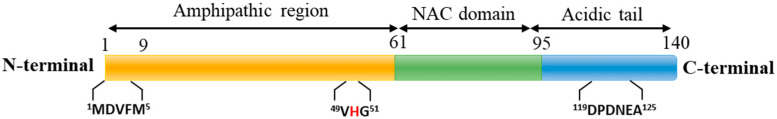
Schematic representation of the full-length αSyn sequence, showing the major regions and some elements of secondary structure.

The protein can be divided into three separate parts: the amphipathic N terminus (residues 1–60), which interacts with lipids; the hydrophobic self-aggregating sequence, also called the non-Aβ component (NAC) because it is a component of the amyloid plaques found in AD patients (residues 61–95); and the acidic C-terminal region (residues 96–140), which plays a crucial role in the chaperone-like activity of αSyn (Figure 1) [21]. 

In its normal, healthy state, αSyn is soluble and it is believed to be involved in regulating neurotransmitter release, maintaining synaptic function, glucose levels, biosynthesis of dopamine, and modulating synaptic vesicle dynamics [22].

However, in PD and DLB, αSyn undergoes a conformational change, adopting a misfolded and aggregated form (Lewy bodies and Lewy neurites). It is still not clear precisely how αSyn aggregates contribute to neurodegeneration, but it is believed that they disrupt cellular processes, impair protein degradation mechanisms, and induce toxicity, leading to neuronal dysfunction and death [21]. Research suggests that genetic and environmental factors contribute to the accumulation and aggregation of αSyn. Mutations or duplications in the SNCA gene, which encodes αSyn, are associated with familial forms of PD, highlighting the significance of this protein in disease development. Autosomal dominant early onset PD is induced as a result of different missense mutations in the αSyn gene (A30P, A53T, and E46K) or as a result of the overexpression of the wild-type αSyn [23]. The αSyn fibrillation occurs through the formation of nuclei from monomers, leading to the formation of β-sheet-aligned, fibrillar aggregates. Recent findings suggest that αSyn fibrils from external regions can be taken up by neuronal cells and serve as seeds for the internal fibrillation of αSyn, leading to neurotoxicity in the neurons. The structural and size differences observed in αSyn fibrils also play a role in determining the toxicity [24]. It is worth noting that fibrils can release prefibrillar oligomers of αSyn that have the ability to effectively traverse neuronal membranes and make them permeable, leading to damage to the cells. Comparatively, short fibrils are more detrimental to neurons than long fibrils, as they possess a greater ratio of fibrillar ends, intensifying their neurotoxicity [25]. The formation of αSyn aggregates is influenced by various factors, including post-translational modifications, pH, polyamines, and the concentration of αSyn [26]. Furthermore, there is evidence that external factors, such as exposure to metal ions or oxidative stress, may trigger the misfolding and aggregation of αSyn. Aggregation rates of αSyn have been demonstrated to be impacted by the presence of metal ions such as Cu^2+^, Zn^2+^, Al^3+^, Fe^3+^, Ca^2+^, and Mg^2+^ [27,28,29,30].

As for copper ions, they can bind to αSyn and influence its behavior and aggregation properties. Because there is evidence of copper imbalances in PD [31], the interaction of αSyn with copper ions could occur. In PD patients, copper levels are elevated in the cerebrospinal fluid and are associated with the presence of Lewy bodies. αSyn has three sites where it can bind copper: residues 1–9, H50, and loop 119–127. αSyn and Cu^2+^ form different complex species: at pH < 6 Cu^2+^ binds to M1 and D2 in a 2N2O (NH_2_, N^−^, COO^−^, O_water_) species, whereas at pH7.4 H50 is involved in forming a 3N1O (NH_2_, N^−^, N_H50_, COO^−^) complex species; a second Cu^2+^ ion binds to H50 in a 2N2O/3N1O (N_H50_, N^−^, N^−^/O, O) species, while the first equivalent occupies the N-terminal binding site (M1 and D2) [32]. Finally, Cu^2+^ also binds to a nonspecific site 3 at the C-terminal region (loop D119-M127). There is also the possibility of ternary species, such as αSyn−Cu^2+^-αSyn′ where the Cu^2+^ ion bridges two distinct protein molecules [32]. In this arrangement, αSyn is bound by its N-terminal part, while αSyn′ is bound by the H50 residue. 

This interaction between αSyn and Cu may also have an impact on the aggregating properties, which are widely recognized as a crucial event in the development of PD. While other metal ions interact at the ^119^DPDNEA^124^ motif, in which D121 acts as the main anchoring site in the C-terminus of αSyn [33], copper binds to the N-terminal region with a moderate affinity and strongly affects the fibrillation process of αSyn. Increasing evidence suggests that the effect of Cu^2+^ on αSyn aggregation in laboratory settings is not solely due to specific binding with histidine or the interaction with negatively charged residues in the C-terminal region of αSyn. The mechanism of aggregation triggered by Cu^2+^ shares common features with the other divalent ions only when the protein is exposed to a millimolar concentration of the metal ion [34]. However, this process shows considerable dissimilarity from the specific binding of Cu^2+^ to the N-terminus of αSyn [34]. The promotion of αSyn amyloid formation by copper is a direct result of the formation of a complex between αSyn and copper (with dissociation constants ranging from 10^−10^ to 10^−9^ M) [34] specifically at the ^1^MDVFM^5^ segment, located in the N-terminal region of the protein. Unlike Aβ and Prp^C^, where the aggregation is influenced by the formation of H-Cu^2+^ complexes, the presence of H50 in αSyn does not impact the aggregation initiated by copper binding at the N-terminal region. This suggests that H50 does not actively participate in the structural and biological events associated with the mechanism of copper-induced αSyn aggregation. The interaction between αSyn and copper is complex and can have different effects on αSyn aggregation [35]. The balance between these effects and the specific conditions under which they occur is still an area of active research. Copper ions can facilitate the transition of αSyn from a monomeric form to aggregated structures, such as oligomers. The presence of annular oligomers during the initial phase of αSyn aggregation has also been reported only in the presence of Cu [36]. Rapid molecular dynamics simulations of αSyn suggest that the presence of Cu(II) ions leads to modifications in the secondary structure pattern of the peptide, resulting in the formation of enhanced and more enduring secondary structural elements like β-strands and hairpins [37]. The aggregated forms of αSyn are believed to be toxic to neurons and contribute to the development and progression of neurodegenerative diseases like PD. In particular, Cu^2+^-promoted αSyn nucleation delays the elongation of fibrils. The interaction between αSyn monomers and Cu^2+^ through macrochelation causes a strain in the conformation of the monomer. This strain disrupts the elongation of fibrils but promotes their nucleation. H50 is situated within the β-sheet-aligned core region of αSyn, and the conformation of the αSyn-Cu^2+^ complex is constrained specifically within residues 1–50. As a result, the structural reorientation of residues 1–50, which is necessary for the assembly onto the αSyn nucleus, is altered [38]. This is further supported by evidence that H50 in mature fibrils cannot coordinate copper [39]. This non-canonical process leads to the formation of shortened αSyn fibrils enriched in β-sheet structures, which are smaller in size (<0.2 μm). These shortened fibrils exhibit rapid transmission and accumulation in neuronal cells, ultimately resulting in neuronal cell death. This is in stark contrast to typical αSyn fibrils, which are larger in size (approximately 1 μm) [38]. TETA (triethylenetetramine) has been demonstrated to mitigate the harmful impact of copper ions on the toxic spread of αSyn fibrils. In a C. elegans model of PD, TETA treatment resulted in the restoration of the organism’s lifespan [40]. These findings highlight TETA as a promising therapeutic approach for PD.

Additionally, the interaction between αSyn and copper can lead to the generation of reactive oxygen species (ROS) through redox reactions. One of the most relevant relations between the formation of copper-αSyn and its toxicity is represented by post-translational modifications induced by metal-induced oxidative stress, copper can also directly or indirectly mediate these post-translational modifications that further alter the structure and function of αSyn, promoting its aggregation and toxicity [41,42].

Understanding the role of copper in αSyn biology may provide insights into the underlying mechanisms of neurodegenerative diseases and could potentially lead to the development of therapeutic strategies targeting this interaction.

### 2.2. Prion Protein

**Figure 2:**
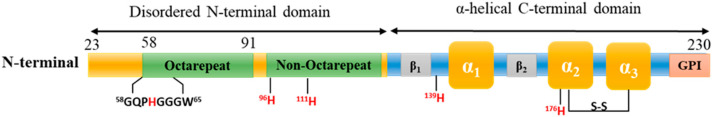
Schematic representation of the full-length sequence of human prion protein, showing the major regions and some elements of secondary structure.

A prion is an infectious, misfolded, and self-replicating protein that can cause neurodegenerative diseases, such as Creutzfeldt–Jakob disease (CJD) in humans or bovine spongiform encephalopathy (BSE) in cattle. Prions do not contain genetic material like viruses or bacteria but can transmit their abnormal conformation to normal proteins, leading to a chain reaction of misfolding and aggregation, ultimately causing damage to the brain and nervous system. Prion diseases are often fatal and difficult to treat [43,44,45]. Prions are composed of an abnormal isoform of a cellular protein called PrP^C^ (Prion Protein). PrP^C^ is a soluble protein with a functional role, consisting of 208 amino acid residues, and is highly expressed in the central nervous system. It comprises two distinct structural regions: an unstructured N-terminal region and a globular C-terminal domain, primarily composed of α-helices. This C-terminal domain is anchored to the pre- and postsynaptic membranes through a GPI (Glycosylphosphatidylinositol) lipid anchor motif (Figure 2) [46].

However, in prion diseases, the misfolded form, known as PrP^Sc^ (Sc for scrapie, a prion disease in sheep), adopts a different conformation with a high content of β-sheet structures.

The misfolded PrP^Sc^ acts as a template, converting the normal PrP^C^ into the abnormal form by inducing its misfolding and aggregation. This process leads to the accumulation of PrP^Sc^ aggregates, which are resistant to proteolytic degradation and form insoluble amyloid fibrils. The exact function of the prion protein remains elusive, and many roles have been hypothesized [46]. Based on its similarity to ZIP family proteins [47], it is believed that the prion protein may be involved in metal homeostasis. Considering the dysregulation associated with prion diseases, this is certainly a hypothesis that is being studied. Based on this, superoxide dismutase activity, transmembrane copper transport, copper buffering, neuronal protection, and neuritogenesis have been proposed as activities of PrP^C^ [47]. Moreover, the key role of copper-binding sites in maintaining the neuritogenesis function of PrP has been recently reported [48]. 

The PrP^C^ protein can bind up to six divalent metal ions, including Cu^2+^, through two distinct domains with different affinities and coordination modes for the metal ion. The coordination properties of PrP are highly dependent on Cu^2+^ concentration, Cu^2+^/protein ratio, and pH. Cu coordination properties of the N-terminal region of human PrP^C^ are excellently reviewed elsewhere [49]. Briefly, the six H residues that act as anchoring sites for Cu ions are: H61, H69, H77, and H85 in the octarepeat (OR) region, and H96 and H111 in the non-OR region. The N-terminal domain contains the OR region, consisting of four tandem PHGGGWGQ repeats, and binds Cu^2+^ with a good affinity (around 0.1 nM). The OR region of PrP^C^ is one example of Cu^2+^ coordination toward the C-terminus of the protein as the P residue near the copper-binding region−PHGGGWSQ−hinders coordination toward the N-terminal region [50]. The non-octarepeat region, located nearby, binds Cu^2+^ ions with weaker affinity, probably exploiting H96 and H111 as ligands (Figure 2). Studies have shown that the OR region can bind up to four Cu^2+^ ions, with the first ion having the highest affinity.

The complex formation of Cu^2+^ with PrP^C^ may influence the interaction of the protein with other systems (proteins, lipids, etc.) through multiple mechanisms. These mechanisms include regulating its localization at lipid rafts, causing conformational changes (such as cis-interdomain interactions) that might be recognized by other proteins, forming ternary protein-Cu^2+^-PrP^C^ complexes, or competing for protein-binding sites [51]. Furthermore, copper may play a crucial role in controlling the infectivity of the prion protein. Specifically, the copper-binding site outside the octarepeat region (95-HNQWNKPSKPKTNLK H-110) could be involved in this ability [52]. Compelling evidence suggests that copper facilitates stabilizing interactions between the N-terminal and C-terminal domains, resulting in a more compact folding of PrP^C^ as reviewed elsewhere. [52]. The functional consequences of this Cu^2+^-mediated structural alteration are yet to be thoroughly explored. However, this interdomain interaction might hold significant relevance in the physiological activity of PrPC [46]. Moreover, a recent study has proposed that Cu^2+^ ions could be important factors in converting the PrP^C^ into amyloid structures of the neurotoxic PrP^Sc^ form. Through CD experiments, it has been observed that copper (particularly at higher copper concentrations) induces the transition of OR peptides from random coil and PPII helix conformations to β-sheet structures [53].

The exact role of copper binding to PrP^C^ and its implications in prion diseases are still areas of active research and investigation. The interaction between copper and PrP may have implications in the misfolding and aggregation of the PrP^C^ and could potentially play a role in the pathogenesis of prion diseases. However, more research is needed to fully understand the significance of copper binding to PrP and its consequences.

### 2.3. 6aJL2

Light-chain amyloidosis, also known as AL amyloidosis, is a rare disease characterized by the abnormal deposition of misfolded light-chain proteins in various organs and tissues throughout the body. It is a type of systemic amyloidosis, where excess light chains produced by abnormal plasma cells misfold and aggregate, forming insoluble amyloid fibrils. These fibrils can deposit in organs such as the heart, kidneys, liver, nervous system, and gastrointestinal tract, leading to organ dysfunction and a wide range of clinical manifestations [54,55].

The symptoms and complications of light-chain amyloidosis can vary depending on the organs affected. Common symptoms may include heart-related issues like heart failure or arrhythmias, kidney dysfunction, liver enlargement, peripheral neuropathy, and gastrointestinal problems [56]. While there are no direct pieces of evidence linking metal ions to the development of this disease, there have been reports indicating that the destabilization of plasma proteins, including LC, could occur in the presence of elevated levels of copper ions.

**Figure 3:**
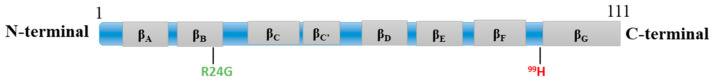
Schematic representation of the primary sequence of the protein 6aJL2-R24G, displaying some elements of the secondary structure.

The main components of the amyloid deposits in AL are primarily derived from fragments of the variable region of the immunoglobulin light chain. Approximately 30% of reported AL cases are associated with λ6 proteins, which has been observed in over 30% of AL patients. Furthermore, it has been noted that 25% of these amyloidogenic proteins have a mutation involving an Arginine to Glycine substitution at position 24. In vitro, studies have demonstrated that this mutation reduces the protein’s stability and increases its tendency to form larger aggregates [57]. Overall, the protein 6aJL2-R24G is composed of eight β-strands (A–C, C′, and D–G) forming a β-sandwich comprising 111 amino acid residues (Figure 3) [58]. Studies have indicated that the protein, in its native or fully unfolded state, does not undergo fibrillar aggregation. This suggests that the formation of aggregates involves the participation of partially unfolded intermediaries as supported by a recent paper [57]. Cu^2+^ has been associated with the development of degenerative diseases, showing affinities similar to those observed for 6aJL2-R24G [59]. Therefore, the binding of Cu^2+^ to 6aJL2-R24G could potentially trigger the aggregation under physiological conditions. In particular, the study revealed that 6aJL2-R24G has the capability to bind Cu^2+^ with submicromolar affinity, and this binding process promotes the formation of protein fibrils at a higher rate as demonstrated by ThT and thermal stability assays. H99 has been indicated as the main interaction site. Moreover, MD simulations of the complexes demonstrated binding site-specific effects, “inducing larger fluctuations of the CDR1 and loop C″ and resulting in increased flexibility and disrupted interactions in critical regions of the molecule [58].

### 2.4. Amyloid-Beta

AD is primarily associated with the accumulation of two types of protein aggregates: β-amyloid (Aβ) plaques and tau tangles. Aβ peptides form plaques outside the neurons whereas Tau isoforms, which are normally involved in stabilizing the structure of neurons, become abnormally phosphorylated and aggregate into tangles within the neurons. The accumulation of Aβ plaques and tau tangles and the interaction between Aβ and tau seem to be related to neuronal dysfunction, leading to the cognitive decline observed in AD [60,61]. Furthermore, disrupted metal homeostasis in the brain and oxidative stress are observed in AD. Notably, Aβ plaques contain higher concentrations of metal ions (Cu, Zn, Fe) compared to normal brain tissue [62]. The binding of metal ions can alter the aggregation of Aβ, leading to disruptions in metalloenzyme activity and promoting the generation of ROS.

The Aβ peptide, derived from the amyloid precursor protein (APP), is produced through cleavage events by α-, β-, and γ-secretases, resulting in predominantly Aβ_1–40_ and Aβ_1–42_ forms [63]. Additionally, truncation at the N-terminus leads to Aβ_3(p)–n_, Aβ_4–n_, and Aβ_11(p)–n_ peptides (p refers to pyroglutamate) found in amyloid deposits [64]. Aβ exists in three forms in the brain: membrane-associated, aggregated, and soluble [63]. In AD, the aggregated and soluble fractions of Aβ increase significantly compared to healthy individuals. Aβ_1–40_ or Aβ_1–42_ undergo on-pathway aggregation, involving primary nucleation, elongation, and plateau phases [65,66]. In particular, Aβ_1–42_ monomers, which are partially folded, form relatively stable oligomers (e.g., pentamers and hexamers) that can further aggregate into protofibrils and fibrils. On the other hand, Aβ_1–40_ initially is a blend of monomers, dimers, trimers, and tetramers, and it undergoes self-assembly into fibrils at a slower rate. Additionally, monomers can transform into nuclei on the surface of fibrils, known as secondary nucleation. Soluble oligomers are currently considered the most toxic species and cause toxicity by interacting with lipid rafts and synaptic receptors on cellular membranes [67,68].

Due to the strong affinity of Aβ peptides for metal ions, the interaction between them and Cu has been extensively studied [69,70,71]. Numerous research groups have put forth various coordination models for Cu^2+^-Aβ complex species based on the data obtained via several techniques such as EPR, CD, NMR, X-ray absorption spectroscopy, and more. Copper complex species of Aβ peptides have been the subject of numerous reviews [71,72,73,74]. In conditions close to physiological pH, the Cu^2+^-Aβ complex exists in two main species known as components I and II. Component II is formed from component I by deprotonating an amide bond in the peptide backbone and subsequently binding it to Cu^2+^. The shift from component I to II occurs at approximately pH 7.8. Both species adopt a distorted square-planar geometry. In component I, Cu^2+^ is involved in a 2N2O coordination mode, equatorially bound to ^1^D, the adjacent CO from the ^1^DA^2^ peptide bond, an N atom from H6, and another N atom from either H13 (component Ia), H14 (component Ib), or both H13 and H14 (component Ic). The apical position can be occupied by an O atom from a water molecule or the carboxylate group of D or E. In component II, the equatorial Cu^2+^ ligands consist of the N-terminal amine, the amide from the ^1^DA^2^ bond, the adjacent CO from the ^2^AE^3^ peptide bond, and one N atom from one of the three H residues. 

Through the integration of capillary electrophoresis studies and the ThT assay, it is possible to confirm different aggregation pathways of Aβ contingent on the ratio of metal-to-peptide [65,75]. When exposed to sub-equimolar and equimolar concentrations of Cu^2+^, Aβ_1–40_ predominantly exhibited linear fibrils, whereas the treatment with 2 equiv of Cu^2+^ resulted in a combination of linear fibrils and amorphous aggregates as demonstrated by AFM. Upon increasing the Cu^2+^-to-Aβ_1–40_ stoichiometry to 6:1, the predominant observation shifted to amorphous aggregates. CD spectroscopy also confirms the changes from random coil structures to anti-parallel β-sheets in the presence of Cu^2+^. Cu^2+^ has the potential to worsen the neurotoxic effects of Aβ since it has been observed that the Cu^2+^-Aβ complex is more toxic than Aβ alone. The binding of Cu to amyloid plaques disrupts the balance of Cu levels between the intracellular and extracellular environments.

Moreover, proposed mechanisms of the higher toxicity involve Cu-catalyzed ROS production and/or its ability to alter the Aβ conformation, thereby promoting the formation of more toxic Aβ aggregates [76]. It has been proposed the ROS production by Cu-Aβ species passes through a low-populated “catalytic in-between state” that is in equilibrium with the resting state of both Cu^+^–Aβ and Cu^2+^–Aβ [77,78,79,80]. This state is responsible for Cu-catalyzed ROS production and contributes to oxidative stress that is another hallmark of AD, as indicated by early changes in neurons and pathological signs of oxidative damage [81,82]. Several studies have shown that the Aβ–Cu^2+^ complex is particularly concerning as it catalytically generates harmful ROS, especially in the presence of cholesterol and vitamin C, resulting in the production of superoxide anion (O_2_^−^), hydrogen peroxide (H_2_O_2_), and hydroxyl radical (·OH) [83,84].

In response to the Cu dyshomeostasis observed in AD and the toxicity triggered by copper-Aβ species, extensive research and clinical trials have been conducted with the primary goal of addressing this issue using copper ionophores or chelators [7,76,85,86,87,88]. Recently, strategies that exploit the coordination sphere of Cu^2+^ bound to Aβ using a chemical reagent by promoting copper–O_2_ chemistry, have been proposed to inhibit Cu^2+^ binding to Aβ and alter the aggregation and toxicity of Aβ [89].

### 2.5. Tau

Tau is a protein with a native unfolded structure and plays a crucial role in the assembly of microtubules. In tauopathies such as AD, Pick’s disease, and progressive supranuclear palsy, tau becomes hyperphosphorylated, leading to the formation of insoluble aggregates called neurofibrillary tangles [90,91]. These disrupt the normal functioning of neurons, impairing their ability to communicate and transport essential nutrients and molecules. As a result, affected neurons may degenerate, leading to progressive neurological dysfunction and cognitive decline. In addition to the harmful effects of aggregated tau, it has been suggested that a potential loss of its normal physiological function could result in microtubule destabilization and impaired axonal transport. 

**Figure 4:**
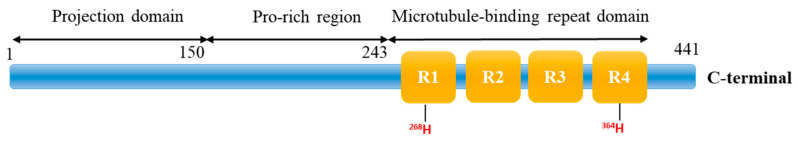
Schematic representation of human tau, showing the main domains.

The full-length human tau protein is generally around 352 to 441 amino acids long, depending on the isoform. Some of the key amino acids and regions in the tau protein include: (i) the N-terminal region, which contains the projection domain, which interacts with microtubules; (ii) the proline-rich region, which contains proline residues that contribute to tau’s conformation and binding capabilities; (iii) microtubule-binding repeats, which consist of four-repeat (4R) or three-repeat (3R) units, which play a role in binding to microtubules; and (iv) the C-terminal region, which contains the region involved in promoting self-assembly and aggregation (Figure 4) [92]. The specific amino acid sequence of tau determines its functions and interactions with other molecules within the cell [93,94,95]. However, due to the complexity of tau isoforms and post-translational modifications, tau’s function and behavior can be highly diverse and regulated in various ways [96]. 

The exact role of copper in tauopathies is still not fully understood, and it is likely to be influenced by various factors, including genetic predisposition, copper levels, and other environmental factors [97]. Moreover, the relationship between copper and tau is just one aspect of the complex mechanisms involved in neurodegenerative diseases. However, there is evidence that copper binds tau, but the formed complex species (binding sites) and the effects of Cu on tau aggregation are controversial in the literature [17,98,99,100,101,102]. Probably, the analysis is complicated by different conditions (pH, equivalents of Cu) and the different fragments of tau that have been studied. Cu^2+^ interacts with full-length microtubule (MT) binding repeats R1 (244–274), R2 (275–305), R3 (306–336), and R4 (337–368), leading to peptide aggregation, fibril formation, and ROS generation for R2 and R3. Ahmadi et al. demonstrated the involvement of H268 in R1 and H363 in R4 in copper binding. Moreover, they revealed through MS studies that while for R1 and R4, metalation was observed leading to the formation of M+Cu and M+2Cu adducts, for R2 and R3, MS results showed the presence of metal complexes related to R2 and R3 dimers [103]. The thiol groups of C residues located in R2 (C291) and R3 (C322) are oxidized in disulfide bonds upon Cu^2+^ complexation. There are notable variations in the capacity of R1-R4 to form aggregates in the presence of Cu^2+^. R1 and R4 exhibited oligomeric aggregates within 3 days, followed by the subsequent formation of larger amorphous aggregates. TEM images revealed that Cu^2+^ played a role in mediating the formation of fibrils and protofibrils for R2 and R3, respectively [103]. 

These findings shed light on the role of Cu^2+^ in various stages of aggregation, inducing conformational changes in MT binding repeats, promoting the dimerization of R2 and R3, forming amorphous aggregates in R1 and R4, and initiating fibrillization in R2 and R3. The results emphasize the involvement of Cu in producing small toxic aggregates that may contribute to neuronal death and ROS formation through the redox chemistry of C291 and C322 [103]. However, as stated before, the high molecular weight of tau, the large number of different post-translational modifications, and the various isoforms make the study of the complex Cu-tau species truly intricate. The same can be said on the effects that copper can have on the function, aggregation, and toxicity of tau. Therefore, much remains to be unveiled about this complex interaction. 

### 2.6. Amylin

Amylin, also known as islet amyloid polypeptide (hIAPP), is a peptide hormone composed of 37 amino acids. The native form of amylin is amidated at the C-terminus and has a C2-C7 disulfide bridge [104,105]. It plays a role in regulating blood sugar levels and appetite, thereby preventing sudden spikes in post-prandial blood glucose levels [106,107]. hIAPP is produced and secreted by the β cells of the pancreas, along with insulin, in response to food intake. Amylin acts as a partner to insulin in controlling glucose metabolism [108]. However, hIAPP cannot be used as a diabetic treatment drug due to its propensity to form cytotoxic fibrils, which have been linked to β-cell degeneration in T2DM [109,110]. The aggregation of amylin is affected by several environmental variables (such as pH, metal ions, and temperature) and components present in pancreatic β-cells [111]. For instance, when the pH of the secretory granule is low (pH 5.5), H18 of hIAPP remains protonated, preventing fibrillation from occurring under these conditions [112]. 

At physiological pH (pH 7.4) and temperature (37 °C), the peptide alone can self-assemble into β-sheet-rich aggregates, leading to the formation of amyloid fibrils, particularly in conditions like type 2 diabetes. The minimal unit for the Cu binding to hIAPP is defined as HSSNN [113,114,115] toward the C-terminal of peptide. Cu^2+^ forms a 3N1O species where it coordinates with the N1 (also known as Nδ) of H18, two deprotonated amides from S19 and S20, and one O atom provided by either the hydroxyl group or the backbone carbonyl of S20. These coordination modes form two sets of three chelate rings with seven, five, and five members each [113]. S20 is also identified as an important residue that stabilizes Cu^2+^ coordination to hIAPP, providing the ligands necessary for forming a stable chelate with two five-membered rings. Further research is needed to fully elucidate the active site environment and the binding interactions in the Cu^2+^-IAPP complex. According to other reports, Cu-hIAPP forms a square-planar complex with a 4N ligating mode at pH 6.0 and above [116] whereas Seal and Dey suggest that the presence of multiple complex species with a 1:1 M/L ratio based on pH, and particularly two of these species, would be physiologically and pathologically relevant [117]. However, Cu^2+^ coordination with hIAPP involves key residues that play a crucial role in the aggregation of hIAPP [118]. H18 serves as the anchoring residue for Cu^2+^ coordination to the disordered peptide and is positioned within the β-sheet structure of the hIAPP fibrils. This binding of Cu^2+^ competes with β-sheet formation, leading to an inhibitory effect on amyloid aggregation. 

Upon binding to amylin, copper stabilizes a set of peptide conformers that would not be capable of undergoing conversion into β-sheet structures, raising the energetic barrier to amyloid fibril formation and effectively inhibiting its fibrillation process. Studies indicate that copper effectively inhibits hIAPP fibrillation in a dose-dependent manner but does not reduce its toxicity [119]. Instead, copper stabilizes hIAPP in certain oligomeric intermediates [120], which exhibit higher toxicity compared to hIAPP fibrils [111]. Analogously to Aβ and prion, it has also been demonstrated that Cu^2+^ ions induce dityrosine cross-linking in hIAPP but not in mIAPP. This effect is enhanced by the addition of H_2_O_2_ [121]. The deposition of amyloidogenic IAPP aggregates, the generation of oxidative stress and the formation of dityrosine species in T2DM patients support the existence of IAPP dimers linked by dityrosine in vivo. 

The combined effects of copper and insulin on hIAPP aggregation have been studied. NMR, fluorescence, CD, AFM, and cell cytotoxicity assay data have shown that copper induces hIAPP to form stable toxic oligomers both in the presence and absence of insulin, inhibiting fibrillation. To be precise, the toxic oligomers formed in the presence of insulin show a slightly higher level of toxicity compared to those generated in the absence of insulin [119]. Finally, it is worth noting that IAPP can simultaneously interact and co-deposit with Aβ_1–40/42_ and tau peptides in the cerebrovascular system and gray matter of aging brains, a phenomenon observed in both AD and T2DM. Traditionally, AD and T2DM have been considered as two separate disorders. Nevertheless, mounting epidemiological, observational, and fundamental molecular research evidence has connected T2DM to an increased risk of AD and vice versa [122]. Copper appears to play a leading role in both pathologies. Therefore, points of intersection between copper interaction and IAPP and Aβ should be actively pursued for the development of effective therapies.

### 2.7. Serum Amyloid A Protein (SSA)

Serum amyloid A (SAA) is a well-conserved family of inflammatory acute-phase proteins, and plays a critical role as a major component in secondary amyloidosis [123]. This condition affects approximately 1% of patients with chronic inflammation, such as those suffering from rheumatoid arthritis and neoplastic diseases [124]. The structure and aggregation of SAA proteins strongly affect the function and pathological implications of these proteins. SAAs are acute-phase reactant proteins, meaning their production increases significantly in response to inflammation or infection. Under certain conditions, such as chronic inflammation or prolonged elevation of SAA levels, the protein can undergo conformational changes and form aggregates, leading to the deposition of amyloid fibrils in tissues [125]. 

Despite the careful cataloging of their sequences and polymorphisms, the three-dimensional structures of SAA proteins, which are small in size, have remained elusive because of their poor water solubility. Lu et al. have reported the crystal structure of SAA1.1 that exists as a hexamer with subunits displaying a four-helix bundle fold stabilized by the interactions present in the C-terminus [124].

Factors such as pH, temperature, and the presence of cofactors can influence the propensity of SAA to aggregate [126,127]. Additionally, genetic mutations in the SAA gene can also increase the risk of amyloidosis [128]. The effects of copper on human SSA1 proteins have not been thoroughly investigated yet; there is only one study concerning the interaction between murine SAA2.2 and metal ions such as copper and zinc. In particular, it was observed that the presence of copper (10–100 µM) alters the equilibrium from hexamer to monomer, while having minimal impact on the stability of the tertiary and secondary structure of SAA2.2 [129]. Since SAA1 and murine SAA2 have some differences in their amino acid sequences, including the conservation of specific residues of H (probably an anchoring site for copper ions, of the three H present in both proteins only the one at position 7 is conserved), it is not easy to predict if copper would have a similar effect on SAA1 as observed in SAA2. 

Further research and experimentation would be needed to investigate the specific impact of copper on SAA1 and whether it induces similar structural changes.

### 2.8. Transthyretin

Transthyretin (TTR) is a protein that exists in both the blood and cerebrospinal fluid. It is primarily synthesized by the liver and acts as a carrier, facilitating the transportation of thyroid hormones and retinol (vitamin A) throughout the body [130,131].

TTR is a tetrameric protein composed of four identical units and has been associated with familial amyloid polyneuropathy (FAP) and senile systemic amyloidosis (SSA) [132]. Indeed, TTR can misfold and aggregate, leading to the formation of amyloid fibrils that can accumulate and damage organs, impairing their normal function. Amyloidosis associated with TTR is known as TTR amyloidosis or ATTR (Amyloid Transthyretin) amyloidosis [130,131].

TTR interacts with copper in certain contexts, and copper can bind to TTR to form copper-TTR complexes. The copper ion is chelated between H88, H90, and D72 on monomer B, and between H90 and D72 on monomer A of TTR. This results in a unique conformation for the stretch of residues 72–92, which differs from that observed in the other TTR-metal complexes [133]. Copper seems to mediate the interaction of TTR with Aβ and could promote the cerebral clearance of Aβ [133].

### 2.9. Neurokinin B

Neurokinin B (NKB) is a decapeptide (DMHDFFVGLM-amide) that plays a significant role in the nervous system [134]. It is a member of the tachykinin family of peptides. NKB is involved in various physiological processes, including the regulation of neurotransmitter release, pain perception, and inflammation. Studies have shown that NKB can activate its specific G protein-coupled receptor known as NK3R, which triggers intracellular signaling pathways leading to various cellular responses [135]. The activation of NK3R by NKB has been linked to its involvement in reproductive functions, neuroendocrine regulation, and behavioral responses. Furthermore, NKB has been implicated in various pathological conditions [136,137]. Dysregulation of NKB signaling has been associated with disorders such as migraines, neurodegenerative diseases, and certain cancers.

NKB has been recognized as a peptide capable of coordinating Cu^2+^ through its N-terminal amino acids even in the presence of competing synaptic cuproproteins like the prion protein. NKB forms a neutral binary complex with Cu^2+^ [CuII(NKB)_2_]. This complex involves two N-terminal amine and two imidazole nitrogen ligands from each NKB molecule, and this binding significantly alters the peptide structure [18]. However, despite these structural changes, It appears that coordination with copper does not alter the ability of NKB to interact with NK3R and does not hinder intracellular calcium release in 1321N1 astrocytoma cells [18]. Copper binding influences the aggregation of NKB.

NKB shows rapid formation of ThT-positive fibrils with a very short lag phase. Jayawardena et al. have suggested that the lack of a significant lag phase for NKB could help limit the generation of toxic oligomers. Moreover, H3 seems to act as the molecular switch regulating fibrillogenesis; H residue is probably involved in π–π stacking interactions with F side chains, leading to a conformation that promotes fibril formation [138]. In the same paper, the role of copper in the fibrillization process is reported. Cu^2+^ inhibits the formation of fibrils in a dose-dependent manner and disassembles preformed NKB fibrils. These findings have led to the hypothesis that copper may play a role in mediating the conformation of the peptide in the synaptic region, where it can reach micromolar concentrations [138] 

Better understanding of the roles and mechanisms of NKB in the nervous system and its interactions with metal ions may provide valuable insights into potential therapeutic strategies for various neurological and endocrine-related conditions. Ongoing research in this area aims to shed more light on the multifaceted functions of Neurokinin B and its implications for human health.

## 3. Conclusions

All the cases reported in this article clearly demonstrate the central role played by copper and the effects it can have, both in modifying the folding of certain proteins and, for example, in regulating their function and toxicity. In some cases, copper has been shown to accelerate aggregation towards fibrils, while in others, it blocks the aggregation process by stabilizing oligomers, which in turn have shown varying degrees of toxicity. Having a clearer understanding of all the complex species involved in these physiological conditions and their “function/toxicity” in the future could enable us to control pathogenic processes, promoting normal physiological functioning. Copper ionophores or, even better, specific copper shuttles could play a fundamental role in countering pathogenic events.

## Data Availability

Not applicable.
